# The emerging role of m^6^A in aortic dissection: from molecular mechanisms to therapeutic potential

**DOI:** 10.3389/fcell.2026.1906074

**Published:** 2026-10-23

**Authors:** Cheng-Qun She, Chong-Zhong Long, Xiao-Lin Lyv, Zheng-Weng Lei, Wen-An Kuang, Hao Deng, Mei-Ling Yi, Shi-Wei Cai, Xiao-Hua Yu, Yuan Peng, Yao-Guang Feng

**Affiliations:** 1 The First Affiliated Hospital, Department of Cardiothoracic Surgery, Hengyang Medical School, University of South China, Hengyang, Hunan, China; 2 Institute of Clinical Medicine, The Second Affiliated Hospital of Hainan Medical University, Haikou, Hainan, China

**Keywords:** aortic aneurysm, aortic dissection, atherosclerosis, hypertension, m^6^A, obesity

## Abstract

Aortic dissection (AD) is an emergent medical condition with a hidden onset and sudden rupture, which usually results in a high mortality rate. N6-methyladenosine (m^6^A) is the most abundant modification in eukaryotic RNA molecules and plays a critical role in regulating RNA metabolism. Dysregulation of m^6^A modification has been linked to a variety of diseases. Interestingly, aberrant expression of m^6^A-associated factors is observed in AD. The m^6^A methylation can promote or inhibit the development of AD by modulating cell death, oxidative stress, macrophage polarization and VSMC phenotype switching, depending on m^6^A-modified transcripts, readers and downstream pathways. In addition, m^6^A exerts a significant effect on the major risk factors for AD, including hypertension, atherosclerosis, obesity and aortic aneurysm. Thus, modulating m^6^A modification by small-molecule inhibitors, natural products or miRNAs may represent a promising approach for developing new therapeutic interventions for AD. In this review, we first give a brief perspective on m^6^A modification and its regulators. Subsequently, we summarize the recent research progress on the role of m^6^A in AD and related-risk factors, with an emphasis on its diagnostic value and therapeutic potential.

## Graphical Abstract

**Figure:**
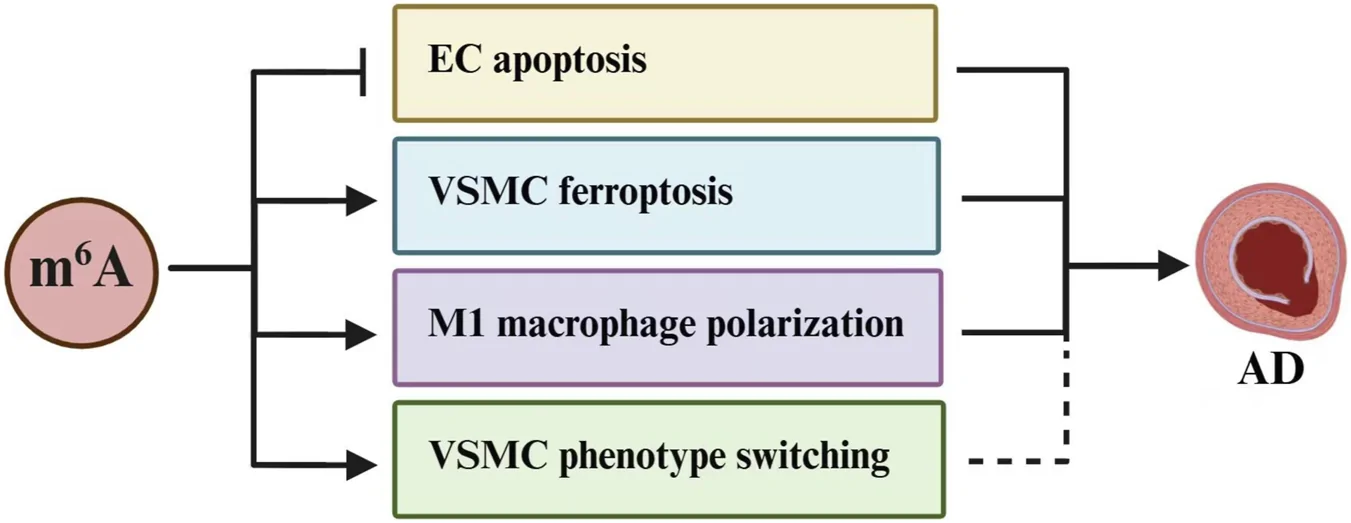

## 1 Introduction

The aortic wall is made up of intima that faces the bloodstream, media and outer adventitia. Aortic dissection (AD) is defined as the separation of the aorta because of a tear within the arterial intima that extends to the media, leading to the entry of blood into media and formation of true and false lumens (Nienaber et al., 2016). It is estimated that the annual incidence of acute AD is 3–5 cases per 100,000 people, a half that of symptomatic aortic aneurysm (Acosta et al., 2006; Olsson et al., 2006). AD is a life-threatening cardiovascular emergency with high mortality during the acute phase and poor prognosis. Based on the anatomical location, AD can be categorized into Stanford type A (or DeBakey type I and type II) involving the ascending aorta, and type B (or DeBakey type IIIa and type IIIb) involving the descending but not the ascending aorta. The etiology of AD is multifactorial and involves a complex interaction between genetic and environmental factors. The risk factors of AD include hypertension, atherosclerosis, obesity, aortic aneurysm and genetic disorders such as Marfan syndrome and Loeys-Dietz syndrome (Zhou et al., 2022a). Its major pathogenesis encompasses inflammatory response, endothelial dysfunction, extracellular matrix degradation (ECM) degradation, oxidative stress, vascular wall cell death, abnormal phenotypic transformation of vascular smooth muscle cells (VSMCs), and angiogenesis (Sayed et al., 2021). Currently, there are limited effective targeted drugs for AD treatment with valid medical evidence. Surgery is considered as the only effective therapeutic method for its clinical management. Thus, it is imperative to strengthen the understanding about the pathophysiology of AD, discover potential biomarkers, develop novel therapeutic interventions.

Epigenetics refers to heritable alterations in gene expression without changing the DNA sequence, which are different from gene mutations (Zhang et al., 2020). The major types of epigenetics include DNA methylation, histone modifications, RNA modifications and chromatin remodeling. With advancements in high-throughput sequencing technologies, nearly 200 types of RNA epigenetic modifications have been identified, with N6-methyladenosine (m^6^A) being the most common, abundant, and highly conserved internal transcript modification in eukaryotic RNAs. The m^6^A modification predominantly occurs in mRNAs, but also exists other types of RNA molecules, such as transfer RNAs (tRNAs), ribosomal RNAs (rRNAs), microRNAs (miRNAs), circular RNAs (circRNAs) and long noncoding RNAs (lncRNAs). The presence of m^6^A on the transcripts exerts a significant effect on RNA fate, including RNA splicing, export, stability, translation, and miRNA biogenesis (Jiang et al., 2021). The m^6^A methylation participates in a wide range of physiological and pathological processes, such as embryonic development, tumorigenesis and neurodegeneration (Li et al., 2023c; Liu et al., 2023; Monteagudo-Garcia et al., 2026). At present, there has been accumulating evidence to indicate a close association of dysregulated m^6^A with cardiovascular diseases (Kumari et al., 2022; Wang et al., 2025a; Yang et al., 2026; Zhang et al., 2026). Interestingly, aberrant m^6^A modification is also involved in the occurrence, progression and rupture of AD (Chai et al., 2021). In this review, we summarized the current knowledge about m^6^A methylation and focused on its role in the pathogenesis of AD, aiming to provide potential approaches for therapeutic interventions and clinical translation.

## 2 Regulators of m^6^A modification

The m^6^A is defined as the methylation modification occurring at the sixth nitrogen atom of adenine within RNA substrates. It is regulated by methyltransferases (writers), demethylases (erasers) and binding proteins (readers). These regulators are responsible for the addition, removal and recognition of m^6^A marks on RNA molecules, respectively (Figure 1; Table 1).

**FIGURE 1:**
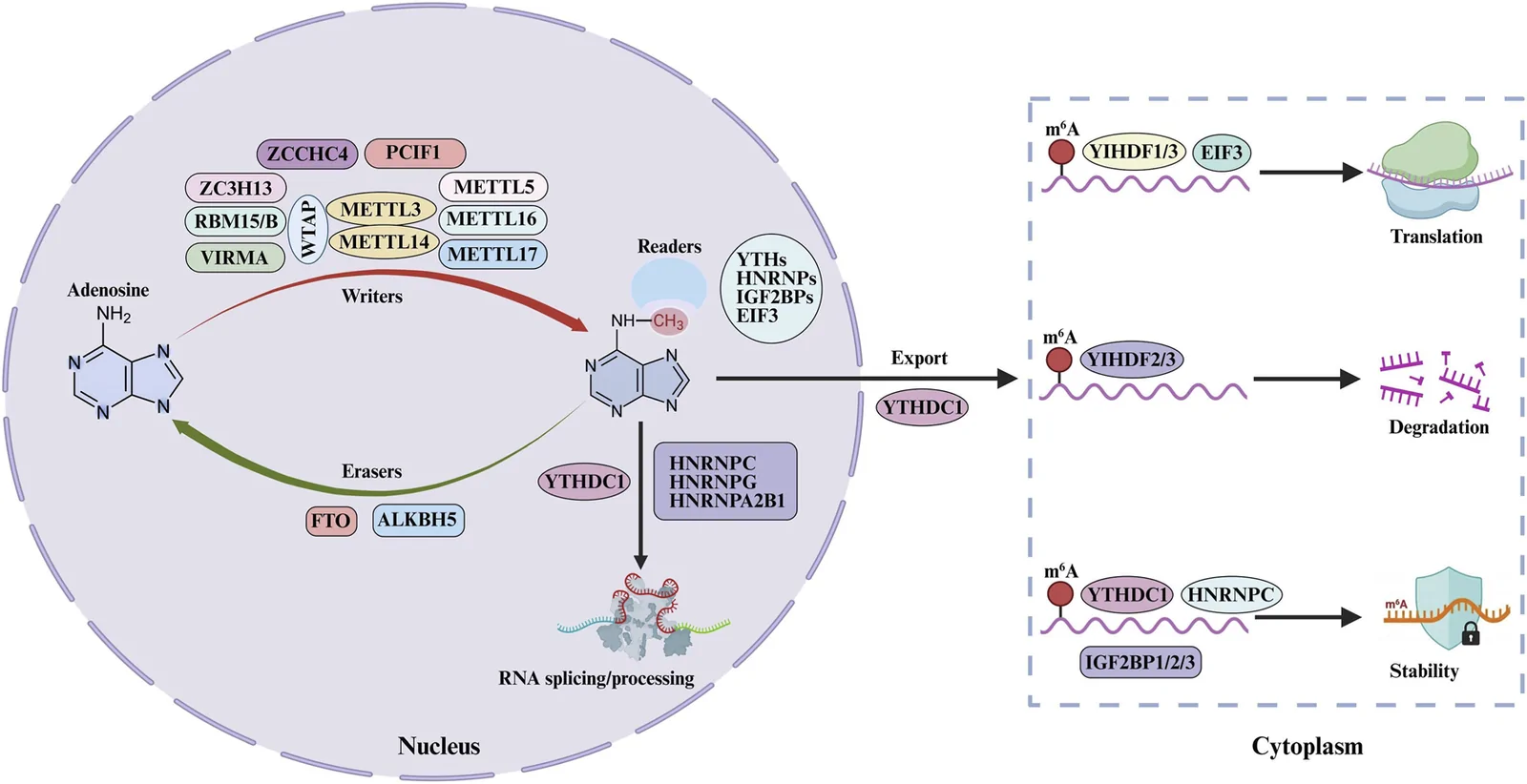
The molecular mechanism and biological functions of m^6^A modification. The m^6^A modification is defined as the methylation of nitrogen at the sixth position of adenosine within RNA molecules. As a reversible epigenetic regulatory mechanism, m^6^A is installed by methyltransferases (writers), removed by demethylases (erasers) and recognized by binding proteins (readers). The common writers encompass the METTL3/METTL14/WTAP complex, METTL5, METTL16, VIRMA, RBM15/RBM15B, ZC3H13, ZCCHC4, PCIF1 and METTL17. The erasers include FTO and ALKBH5. After recognition by EIF3, YTHs, HNRNPs or IGF2BPs, m^6^A exerts a significant effect on various aspects of RNA metabolism, including splicing, transport, localization, degradation and translation.

**TABLE 1:** Biological functions of m^6^A regulators in RNA metabolism.

Types	Regulators	Functions	References
m^6^A writers	METTL3	Catalyzing m^6^A modification	Liu et al. (2014)
​	METTL14	Helping METTL3 to recognize the substrates	Liu et al. (2014)
​	WTAP	Contributing to nuclear localization of the METTL3/METTL14 heterodimer	Ping et al. (2014)
​	METTL5	Catalyzing m^6^A modification in cooperation with TRMT112	van Tran et al. (2019); Dai et al. (2023)
​	METTL16	Catalyzing m^6^A modification	Han et al. (2023); Sun et al. (2023); Ju and Tomita. (2025)
​	KIAA1429/VIRMA	Recruiting the METTL3/METTL14/WTAP complex to guide region-selective m^6^A methylation	Yue et al. (2018)
​	RBM15/RBM15 B	Binding to the m^6^A complex and recruiting it to specific RNA sites	Patil et al. (2016)
​	ZC3H13	Bridging WTAP to the mRNA-binding factor Nito	Knuckles et al. (2018)
​	ZCCHC4	Catalyzing m^6^A modification of human 28 S rRNA and a subset of mRNAs	Ma et al. (2019)
​	PCIF1	Catalyzing m^6^A modification of capped mRNAs	Sendinc et al. (2019); Sugita et al. (2021)
​	METTL17	Catalyzing m^6^A modification of mitochondrial RNAs	Li et al. (2024a)
m^6^A erasers	FTO	Eliminating m^6^A modification	Fu et al. (2014)
​	ALKBH5	Eliminating m^6^A modification	Toh et al. (2020)
m^6^A readers	YTHDF1	Promoting mRNA translation	Zaccara et al. (2019)
​	YTHDF2	Enhancing mRNAs degradation	Zaccara et al. (2019)
​	YTHDF3	Promoting mRNA translation and degradation by interacting with YTHDF1 and YTHDF2	Shi et al. (2017)
​	YTHDC1	Contributing to RNA splicing, transport and stabilization	Xiao et al. (2016)
​	YTHDC2	Promoting mRNA translation	Wu et al. (2024b)
​	EIF3	Promoting mRNA translation	Meyer et al. (2015)
​	HNRNPA2B1	Promoting alternative splicing and miRNA maturation	Alarcon et al. (2015)
​	HNRNPC	Facilitating RNA stabilization and alternative splicing	Liu et al. (2025a); Xiong et al. (2025)
​	HNRNPG	Mediating pre-mRNA processing	Liu et al. (2017); Zhou et al. (2019)
​	IGF2BP1/2/3	Increasing RNA stability	Duan et al. (2024)

### 2.1 Writers

In mammals, the most well-investigated m^6^A methyltransferase is the methyltransferase-like protein 3 (METTL3)/METTL14/Wilms tumor 1-associated protein (WTAP) complex. Among this complex, METTL3 functions as a catalytic subunit, and METTL14 can enhance substrate recognition and RNA binding (Liu et al., 2014). A combination of METTL3 and METTL14 has a higher methyltransferase activity than METTL3 or METTL14 alone (Liu et al., 2014). WTAP is regarded as a regulatory subunit, and contributes to the nuclear localization of the METTL3/METTL14 heterodimer (Ping et al., 2014). METTL5 forms a heterodimeric complex with TRMT112 to catalyze the m^6^A modification at a specific site on human 18S ribosomal RNA (rRNA), thereby promoting ribosome synthesis and mRNA translation (van Tran et al., 2019; Dai et al., 2023). METTL16, another m^6^A methyltransferase, contains an N-terminal methyltransferase domain and two C-terminal RNA-binding domains. Interestingly, U6 snRNA and several mRNAs have been verified to be directly methylated by METTL16 (Han et al., 2023; Sun et al., 2023; Ju and Tomita, 2025). Besides the mentioned above, several m^6^A methyltransferases have been identified. For instance, Vir-like m^6^A methyltransferase associated protein (VIRMA), also called KIAA1429, is the largest component of the m^6^A methyltransferase complex. It can recruit the METTL3/METTL14/WTAP complex to guide region-selective m^6^A methylation in 3′-untranslated region and near stop codon, which is correlated with alternative polyadenylation (Yue et al., 2018). RNA-binding motif protein 15 (RBM15) and its paralogue RBM15B bind to the m^6^A complex and recruit it to specific sites in RNAs (Patil et al., 2016). Zinc finger CCCH domain-containing protein 13 (ZC3H13) promotes m^6^A installation by bridging WTAP to the mRNA-binding factor Nito (Knuckles et al., 2018). Zinc finger CCHC-type containing 4 (ZCCHC4) predominantly methylates human 28S rRNA and also interacts with a subset of mRNAs (Ma et al., 2019). Phosphorylated C-terminal domain-interacting factor 1 (PCIF1) catalyzes only m^6^A modification of capped mRNAs and has not impact on internal m^6^A methylation (Sendinc et al., 2019; Sugita et al., 2021). This suggests that PCIF1 serves as a cap-specific m^6^A methyltransferase. METTL17, which is located in mitochondria through its N-terminal targeting sequence, has been reported to mediate the m^6^A methylation of mitochondrial RNAs and plays a significant role in regulating the expression of mitochondrial protein-coding genes (Li et al., 2024a). Of note, METTL17 can also mediate other RNA methylation modifications such as 3-methylcytidine, 4-methylcytosine, 5-methylcytosine and N7-methylguanosine, indicating METTL17 as a pan-methyltransferase (Li et al., 2024a).

### 2.2 Erasers

Contrary to methyltransferases, demethylases can eliminate the m^6^A modifications from RNA molecules, rendering the process reversible. Demethylated sites lose their ability to combine with reader proteins, thereby affecting RNA splicing, transport, stability and translation. Fat mass and obesity-associated protein (FTO) is the first identified enzyme responsible for m^6^A demethylation. It can demethylate m^6^A through an oxidative reaction, a catalytic process that is dependent on Fe^2+^ and α-ketoglutarate. FTO first oxidizes m^6^A on RNAs stepwise to form N^6^-hydroxymethyladenosine (hm^6^A). Then, FTO further oxidizes hm^6^A in the same manner to generate N^6^-formyladenosine (f^6^A) (Fu et al., 2014). Finally, both hm^6^A and f^6^A are converted back to adenosine through spontaneous or enzyme-catalyzed hydrolysis (Fu et al., 2013). AlkB homolog 5 (ALKBH5) is another m^6^A demethylase and belongs to the AlkB subfamily. Unlike FTO, ALKBH5 catalyzes the direct conversion of m^6^A to adenosine, with rapid release of formaldehyde (Toh et al., 2020). It is still unclear, however, whether other factors are involved in m^6^A demethylation besides FTO and ALKBH5. Identification of new m^6^A demethylases will not only deepen our understanding of RNA epigenetic regulation but also help develop m^6^A-based therapies.

### 2.3 Readers

Binding proteins, also known as readers, are a group of proteins that directly recognize and interact with m^6^A-modified sites on RNA molecules and thereby determine RNA fate. The first identified and most comprehensively investigated m^6^A readers are YTH domain family (YTHDF) proteins, including YTHDF1, YTHDF2, YTHDF3, YTHDC1 and YTHDC2. YTHDF1 was shown to increase the translation efficiency of mRNAs modified by m^6^A, while YTHDF2 promotes the degradation of m^6^A-modified mRNAs, ensuring dynamic modulation of gene expression (Zaccara et al., 2019). YTHDF3 cooperates with YTHDF1 and YTHDF2 to facilitate mRNA translation and decay, respectively (Shi et al., 2017). YTHDC1 contributes to RNA splicing, transport and stabilization, while YTHDC2 promotes target mRNA translation and protein expression (Xiao et al., 2016; Wu et al., 2024b). Non-YTHDF m^6^A readers comprise eukaryotic initiation factor 3 (EIF3), heterogeneous nuclear ribonucleoprotein (HNRNP) family (HNRNPA2B1, HNRNPC, and HNRNPG) and insulin-like growth factor 2 mRNA-binding protein (IGF2BP) family (IGF2BP1, IGF2BP2 and IGF2BP3). It has been demonstrated that EIF3 interacts with the m^6^A sites on 5′-untranslated region of mRNAs and then recruits the 43S complex to initiate protein translation in a cap-independent manner (Meyer et al., 2015). HNRNPA2B1 can bind to m^6^A marks in nuclear transcripts and primary miRNAs to promote alternative splicing and miRNA maturation, revealing HNRNPA2B1 as a nuclear reader of m^6^A, revealing HNRNPA2B1 as a nuclear reader of m^6^A (Alarcon et al., 2015). HNRNPC stabilizes m^6^A-modified RNAs and contributes to alternative splicing (Liu et al., 2025a; Xiong et al., 2025). HNRNPG binds directly to m^6^A-methylated mRNAs using the Arg-Gly-Gly motifs within its C-terminal low-complexity region and plays an important role in pre-mRNA processing (Liu et al., 2017; Zhou et al., 2019). IGF2BP1, IGF2BP2 and IGF2BP3 can enhance the stability of m^6^A-modified RNAs, leading to upregulation of target gene expression (Duan et al., 2024).

## 3 Detection methodologies of m^6^A modification

A variety of techniques have been developed to characterize m^6^A modification. For instance, m^6^A RNA immunoprecipitation followed by sequencing (MeRIP-seq), also known as m^6^A-seq, is used widely to measure transcriptome-wide m^6^A mapping. This method utilizes anti-m^6^A antibody to immunoprecipitate methylated RNA fragments and then performs next-generation sequencing, thereby enabling the identification of m^6^A peaks across transcripts (Dominissini et al., 2012). It is worth noting that MeRIP-seq only provides the limited resolution (∼100–200 nt) and may not examine low-abundance m^6^A sites. The m^6^A individual-nucleotide resolution crosslinking and immunoprecipitation (miCLIP) uses ultraviolet crosslinking to covalently attach antibodies to RNA, creating mutations or truncations during reverse transcription that can identify precisely m^6^A-modified sites (Linder et al., 2015). Despite miCLIP can offer single-nucleotide resolution for m^6^A mapping throughout the transcriptome, it is expensive and labour-intensive. Liquid chromatography-tandem mass spectrometry (LC-MS/MS) belongs to a quantitative method to detect global m^6^A levels and cannot determine m^6^A-modified sites (Vignon et al., 2023). In addition, nanopore-based approaches are able to measure and quantify m^6^A modification directly from native RNA molecules (Liu et al., 2019). A major limitation for this technology is relatively low sensitivity for low-abundance transcripts.

## 4 Role of m^6^A modification in AD

AD is regarded as a very serious vascular disease that requires immediate treatment. Dysregulation of m^6^A regulators has been shown to significantly affect AD progression through multiple mechanisms, such as cell death, oxidative stress, macrophage polarization and VSMC phenotype switching (Figure 2; Table 2).

**FIGURE 2:**
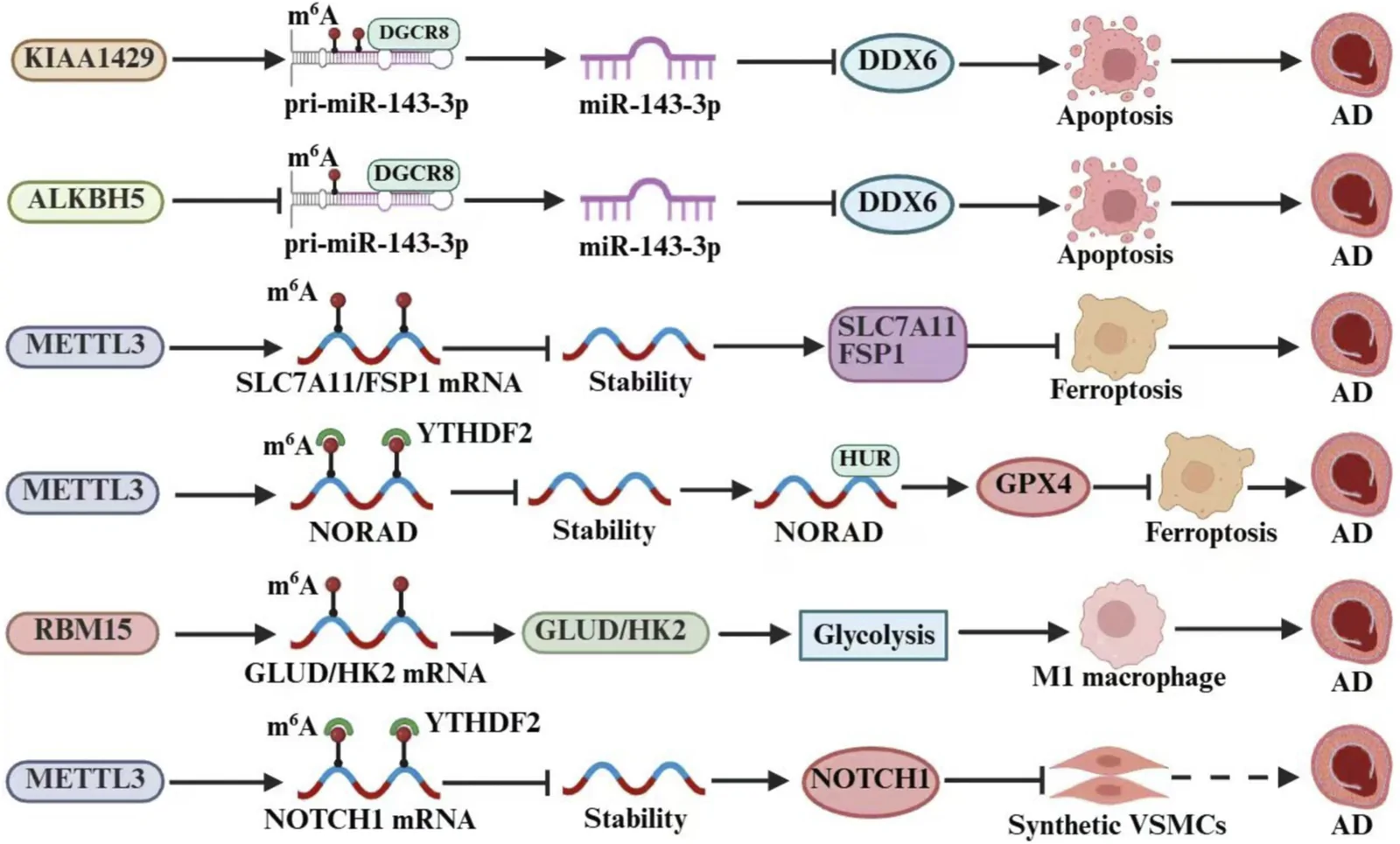
The impact of m^6^A regulators on AD. KIAA1429 inhibits EC apoptosis and AD development through the pri-miR-143-3p/miR-143-3p/DDX6 signaling cascade, whereas ALKBH5 exhibits an opposite effect. METTL3-mediated m^6^A methylation down-regulates the expression of SLC7A11, FSP1 and NORAD to stimulate HASMC ferroptosis and consequently exacerbate AD. Additionally, METTL3 cooperates with YTHDF2 to destabilize NOTCH1 mRNA and facilitate VSMC phenotype switching. In addition, RBM15 was shown to promote glycolysis, M1 macrophage polarization and AD progression by methylating GLUT1 and HK2 transcripts.

**TABLE 2:** Experimentally verified m^6^A regulators and their role in AD.

Regulators	Expression	Targets	Readers	Cell types	Effects on AD	References
KIAA1429	Down	Pri-miR-143–3p	Not involved	VSMCs and ECs	Protection	Wang et al. (2021a)
ALKBH5	Up	Pri-miR-143–3p	Not involved	VSMCs and ECs	Promotion	Wang et al. (2021a)
METTL3	Up	SLC7A11 and FSP1	Not involved	VSMCs	Promotion	Li et al. (2022)
​	Not involved	NORAD	YTHDF2	VSMCs	Promotion	Liao et al. (2024)
​	Not involved	NOTCH1	YTHDF2	VSMCs	Not involved	Yang et al. (2023a)
RBM15	Up	GLUD and HK2	Not involved	Macrophages	Promotion	Tang et al. (2024b)

### 4.1 Cell death

It is well known that apoptosis is a programmed cell death form characterized by chromosomal concentration, cell shrinkage, DNA degradation and apoptotic body formation, without eliciting inflammatory response. Nowadays, there has been a large body of evidence supporting the apoptosis of VSMCs and endothelial cells (ECs) as a contributor to AD (Zhang et al., 2022; Zhao et al., 2023; Liu et al., 2024). By using high-throughput sequencing technologies, Zhou and colleagues identified 195 m^6^A peaks between the aortic media tissue samples obtained from AD patients and normal controls, corresponding with 179 genes (35 hypermethylated and 144 hypomethylated) (Zhou et al., 2021). The hypermethylated genes are predominantly enriched in the processes associated with extracellular fibril organization, whereas the hypomethylated genes primarily participate in cell death regulation (Zhou et al., 2021). In the aorta isolated from AD patients, KIAA1429 expression is significantly decreased, which is accompanied by an obvious increase of ALKBH5 amount (Wang et al., 2021a). Moreover, lentivirus-mediated overexpression of KIAA1429 promotes the conversion of pri-miR-143-3p to miR-143-3p in a m^6^A-dependent manner by interacting with DiGeorge syndrome critical region gene 8 (DGCR8), which in turn down-regulates DEAD/H-box RNA helicase 6 (DDX6) expression, stimulates VSMC proliferation, hinders EC apoptosis, and alleviates AD in angiotensin II (AngII)-infused mice (Wang et al., 2021a). Nevertheless, an opposite effect appears in response to ALKBH5 overexpression (Wang et al., 2021a). These results establish a novel link of m^6^A to AD. Given that KIAA1429 and ALKBH5 play an important role in regulating cell apoptosis (Liu and Liu, 2023; Wang et al., 2025b), it is also possible that these two m^6^A-associated enzymes affect AD progression by modulating VSMC apoptosis. Further research is required to verify this possibility.

Ferroptosis is a novel cell death manner characterized by iron overload and lipid peroxidation (Dixon and Olzmann, 2024). It has been reported that the expression levels of ferroptosis markers, such as heme oxygenase 1, acyl-CoA synthetase long-chain family member 4 and 4-hydroxynonenal, are significantly increased in the aortic tissue from AD patients and β-Aminopropionitrile (BAPN)-challenged mice (Qi et al., 2024). Induction of ferroptosis in VSMCs and ECs can promote AD development in animal models (Tao et al., 2024; Feng et al., 2025). Conversely, inhibition of ferroptosis by small molecules BRD4770 or UAMC-3203 protects against AD (Chen et al., 2022b; Fu et al., 2026). Thus, ferroptosis functions as a key driver of AD and is considered as a novel therapeutic target.

The m^6^A modification has been reported to regulate ferroptosis in multiple cell types, such as alveolar epithelial cells (Wu et al., 2024a), cardiomyocytes (Yang et al., 2024), and gastric cancer cells (Yang et al., 2022). Interestingly, METTL3 expression is significantly upregulated in the aorta of Stanford type A AD patients and shows a negative correlation with the levels of solute carrier family 7 member 11 (SLC7A11) and ferroptosis suppressor protein 1 (FSP1), two key regulatory proteins of ferroptosis (Li et al., 2022). Moreover, overexpression of METTL3 accelerates the mRNA decay of SLC7A11 and FSP1 in an m^6^A-dependent manner, which promotes ferroptosis of human arterial smooth muscle cells (HASMCs) and aggravates AD in BAPN-treated mice (Li et al., 2022). Non-coding RNA activated by DNA damage (NORAD) is a lncRNA that is essential for genome stability. Recently, Liao et al. reported that METTL3 mediates the m^6^A modification of NORAD, leading to down-regulation of its expression in an YTHDF2-dependent manner and development of AD (Liao et al., 2024). Mechanistically, NORAD directly interacts with human antigen R (HUR), which increases glutathione peroxidase 4 (GPX4) mRNA stability, upregulates GPX4 expression, and eventually suppresses AngII-induced ferroptosis of HASMCs (Liao et al., 2024). These results derived from human observational data, *in vitro* experiments and animal models indicate a close association of METTL3-mediated m^6^A modification with AD. Targeting METTL3 could represent a potential therapeutic option for the treatment of AD.

Pyroptosis is an active and ordered form of programmed cell death characterized by the activation of nucleotide-binding oligomerization domain-like receptor protein 3 (NLRP3) inflammasome and abundant release of pro-inflammatory cytokines (Bai et al., 2025b). Like apoptosis and ferroptosis, pyroptosis functions as a contributor to AD (Meng et al., 2026). Several lines of evidence have demonstrated that dysregulated m^6^A methylation is associated with immune cell infiltration in human AD (Guo et al., 2022; Yin et al., 2022). However, it is still unclear whether m^6^A modification affects vascular inflammation during AD by regulating pyroptosis of vascular wall cells.

As a form of regulated necrotic cell death, necroptosis is activated under apoptosis-deficient conditions and exhibits characteristics of both necrosis and apoptosis (Dai et al., 2021). In the aortic tissue isolated from Stanford type A AD patients, the necroptosis pathways are considerably enriched and activated (Liu et al., 2022). It has been demonstrated that the m^6^A modification plays an important role in regulating necroptosis in multiple cell types, including cardiomyocytes (Jia et al., 2024), neurons (Wang et al., 2026), and alveolar type II epithelial cells (Wu et al., 2026). Nevertheless, there is a lack of the association of m^6^A and necroptosis in the context of AD. Future studies are required to explore the impact of m^6^A on vascular wall cell necroptosis during AD.

### 4.2 Oxidative stress

Oxidative stress, which is derived from an imbalance between reactive oxygen species (ROS) generation and the available antioxidant defense system, can cause oxidative damage of cells and tissues (Hassan et al., 2024). There is considerable evidence demonstrating that oxidative stress can stimulate VSMC apoptosis, enhance ECM synthesis, promote macrophage infiltration, and impair endothelial function, all of which contribute to the pathological remodeling of aortic wall and subsequent development of AD (Xu et al., 2024a; Zhang et al., 2025d). Of note, m^6^A is also involved in the regulation of oxidative stress (Zhu et al., 2024; Jiang et al., 2025). For instance, decreased YTHDC1 expression is observed in the aorta obtained from AD patients, and knockdown of YTHDC1 facilitates ROS production and inhibits the expression of superoxide dismutase 2 in human umbilical vein endothelial cells (HUVECs) (Yin et al., 2023). Thus, the human tissue evidence and *in vitro* experimental results indicate that YTHDC1 may have a potential protective effect on AD. However, whether m^6^A-modified enzymes affect AD development by regulating oxidative stress is still unclear and needs to be investigated.

### 4.3 Macrophage polarization

As the important immune cells in innate immunity, macrophages are divided into two major cell subpopulations: classical activated M1 type and alternative activated M2 type. M1 macrophages, which are predominantly activated by lipopolysaccharide and interferon-γ, can generate a variety of pro-inflammatory cytokines such as interleukin (IL)-1β, IL-6 and tumor necrosis factor-α. In contrast, M2 macrophages are induced by IL-4 and IL-13, and secrete anti-inflammatory cytokines including IL-10, transforming growth factor-β and arginase 1 (Luo et al., 2024). The imbalance between M1 macrophages and M2 macrophages has been linked to the onset and development of many diseases (Wang et al., 2019; Bai et al., 2025a). Currently, it is generally accepted that macrophages are polarized towards the M1 phenotype during AD (Zhang et al., 2024; Wang et al., 2025d). By using the GSE52093 and GSE147027 datasets, Yin et al. found that 279 m^6^A-related genes are differentially expressed between AD and healthy subjects, and the hub genes are correlated with M2 macrophages (Yin et al., 2022). Another study showed that RBM15 is highly expressed in aortic tissue of AD patients, along with increased infiltration of M1 macrophages (Tang et al., 2024b). Further research demonstrated that overexpression of RBM15 enhances glycolysis by up-regulating glucose transporter type 1 (GLUT1) and hexokinase 2 (HK2) expression in an m^6^A-dependent manner, and then promotes macrophage polarization by shifting anti-inflammatory M2 macrophages to pro-inflammatory M1 macrophages, thereby contributing to AD progression in a rat model (Tang et al., 2024b). These *in vitro and in vivo* data suggest that RBM15 serves as a contributor to AD by facilitating macrophage polarization towards the M1 phenotype. The therapeutic approaches aiming to inhibit RBM15-mediated m^6^A modification could be valuable for reducing AD formation and rupture.

### 4.4 VSMC phenotype switching

VSMCs are predominantly located in the medial layer of the arterial wall and possess remarkable phenotypic plasticity. In a quiescent state, VSMCs in mature arteries exhibit a contractile phenotype and play a central role in maintaining normal structure and function of the vessels. Under stress, the contractile phenotype of VSMCs can be transformed into a synthetic phenotype. Synthetic VSMCs are able to abundantly secrete matrix metalloproteinases to degrade ECM components, leading to aberrant proliferation and migration of cells (Cao et al., 2022). Accumulating evidence has demonstrated that VSMC phenotype switching is a critical contributor to the formation and rupture of AD (Leng et al., 2025; Liu et al., 2026). Notch homolog 1 (NOTCH1) is down-regulated in medial VSMCs of aortic tissue from AD patients and has been reported to restrain AD development in animal models (Zou et al., 2012; Zhou et al., 2022b). Of note, Yang and colleagues found that METTL3 overexpression in HASMCs dramatically decreases NOTCH1 mRNA stability by recruiting YTHDF2 in an m^6^A-dependent manner, leading to down-regulation of NOTCH1, α-smooth muscle actin, smooth muscle protein 22-α and calponin expression as well as increased cell proliferation (Yang et al., 2023a). This functional study performed in cultured cells reveals that METTL3 is able to promote the transition of HASMCs from a primary contractile phenotype to a synthetic phenotype. Therefore, in addition to induction of VSMC ferroptosis, stimulating VSMC phenotypic transformation is another important mechanism by which METTL3 aggravates AD.

Taken together, m^6^A can both promote and inhibit AD development. This dual role may depend on m^6^A-modified enzymes, reader availability, RNA substrates, cell types, or RNA localization. Among m^6^A regulators, only the influence of METTL3 on AD has been supported more explicitly by the corresponding independent studies. Moreover, the majority of mechanistic studies rely on AngII or BAPN-induced mouse models and *in vitro* VSMC experiments. Human validation studies are relatively scarce and involves limited sample sizes.

## 5 The effects of m^6^A modification on AD major risk factors

Hypertension, atherosclerosis, obesity and aortic aneurysm are regarded as the most common risk factors for AD (Zhou et al., 2022a). The m^6^A modification not only has a direct effect on AD but also displays a close association with hypertension (Figure 3), atherosclerosis (Figure 4), obesity (Figure 5) and aortic aneurysm (Figure 6).

**FIGURE 3:**
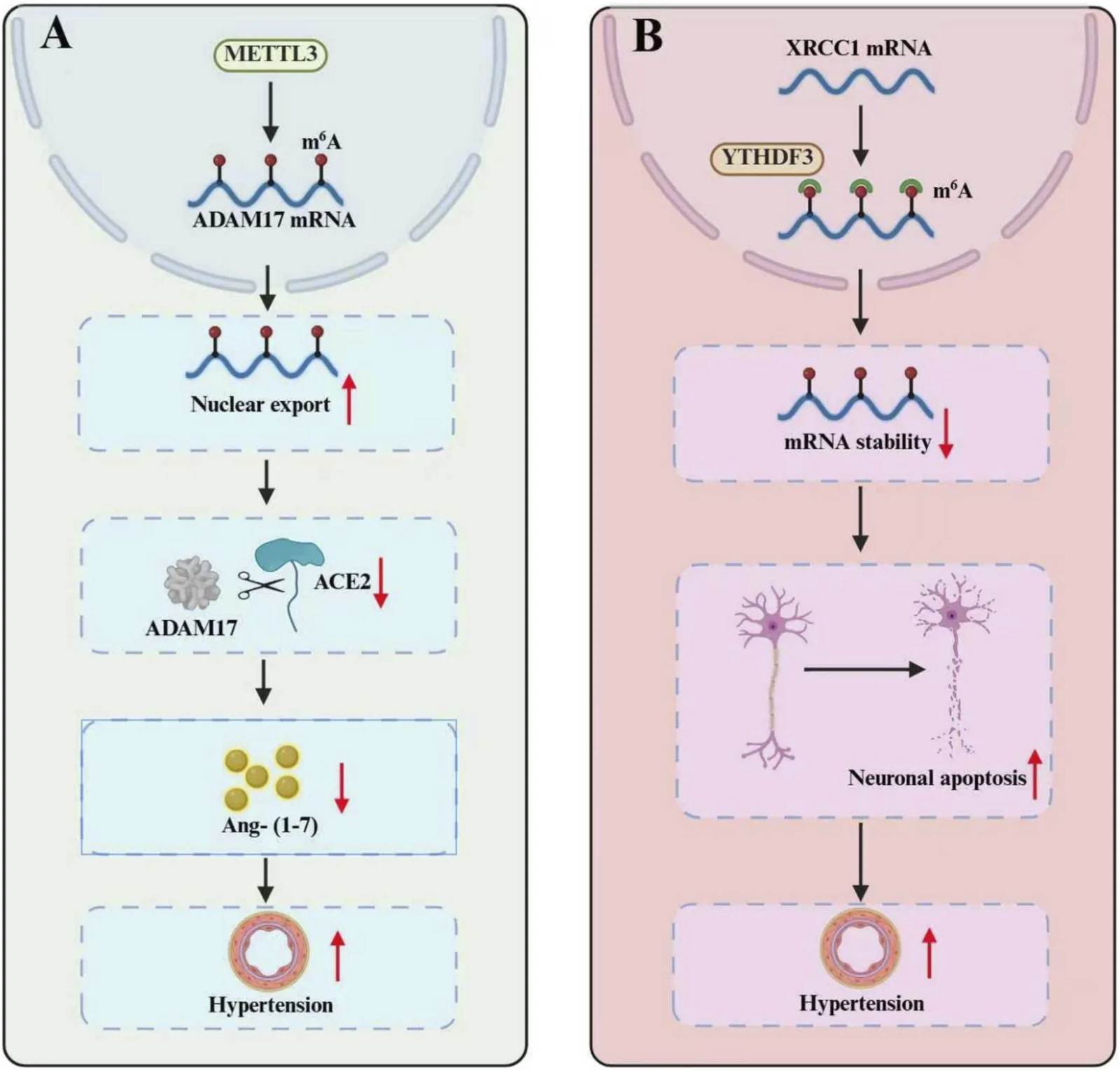
A schematic diagram showing the association of m^6^A with hypertension. **(A)** METTL3 catalyzes the m^6^A modification of ADAM17 mRNA, leading to its transport from the nucleus to the cytoplasm. Then, ADAM17 mediates the cleavage of ACE2 to inhibit Ang-(1–7) biosynthesis and promote hypertension development. **(B)** YTHDF3 binds to m^6^A-modified sites on XRCC1 mRNA, which in turn decreases its stability, stimulates neuronal apoptosis and aggravates hypertension.

**FIGURE 4:**
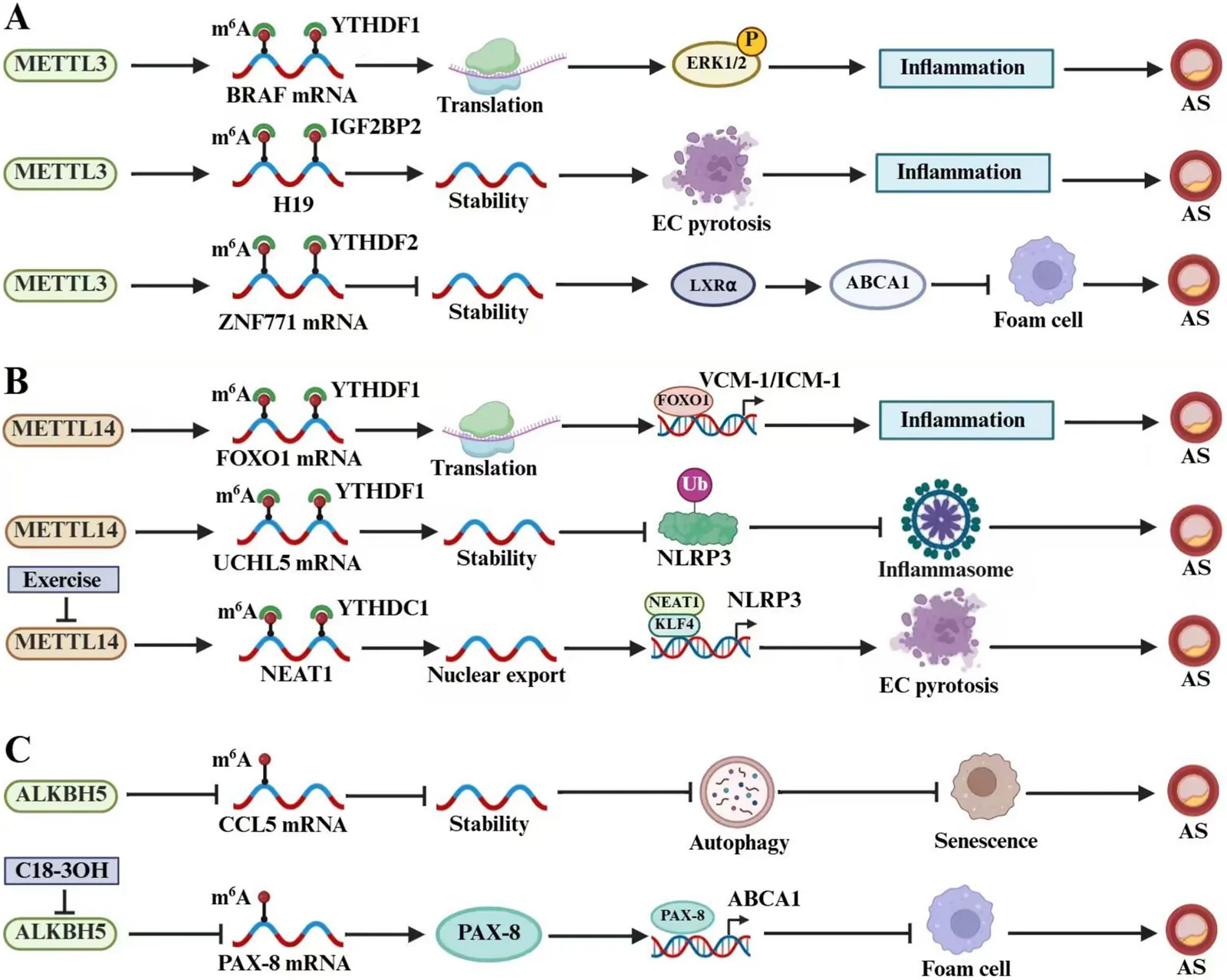
The m^6^A modification affects atherosclerosis development through multiple signaling pathways. **(A)** METTL3-mediated m^6^A methylation of BRAF and H19 transcripts can promote vascular inflammation and atherosclerosis development. In addition, METTL3 decreases ZNF771 mRNA stability by recruiting YTHDF2, which inhibits the LXRα/ABCA1 signaling pathway and aggravates atherosclerosis. **(B)** METTL14 works in concert with YTHDF1 to enhance FOXO1 mRNA translation, thereby facilitating VCAM-1 and ICAM-1 transcription and atherogenesis. METTL14/YTHDF1-mediated m^6^A modification enhances UCHL5 mRNA stability to activate NLRP3 inflammasome and aggravate atherosclerosis. Exercise inhibits METTL14/YTHDC1- mediated m^6^A modification of NEAT1 transcripts, which attenuates its nuclear export, suppresses KLF4-induced NLRP3 transcription, and alleviates endothelial pyroptosis and atherosclerosis. **(C)** ALKBH5-mediated m^6^A demethylation stabilizes CCL5 mRNA, which inhibits autophagy, promotes macrophage senescence and accelerates atherogenesis. Additionally, C18-3OH increases PAX-8 expression by inhibiting ALKBH5-mediated m^6^A demethylation, thereby facilitating ABCA1 transcription and protecting against atherosclerosis. AS: atherosclerosis.

**FIGURE 5:**
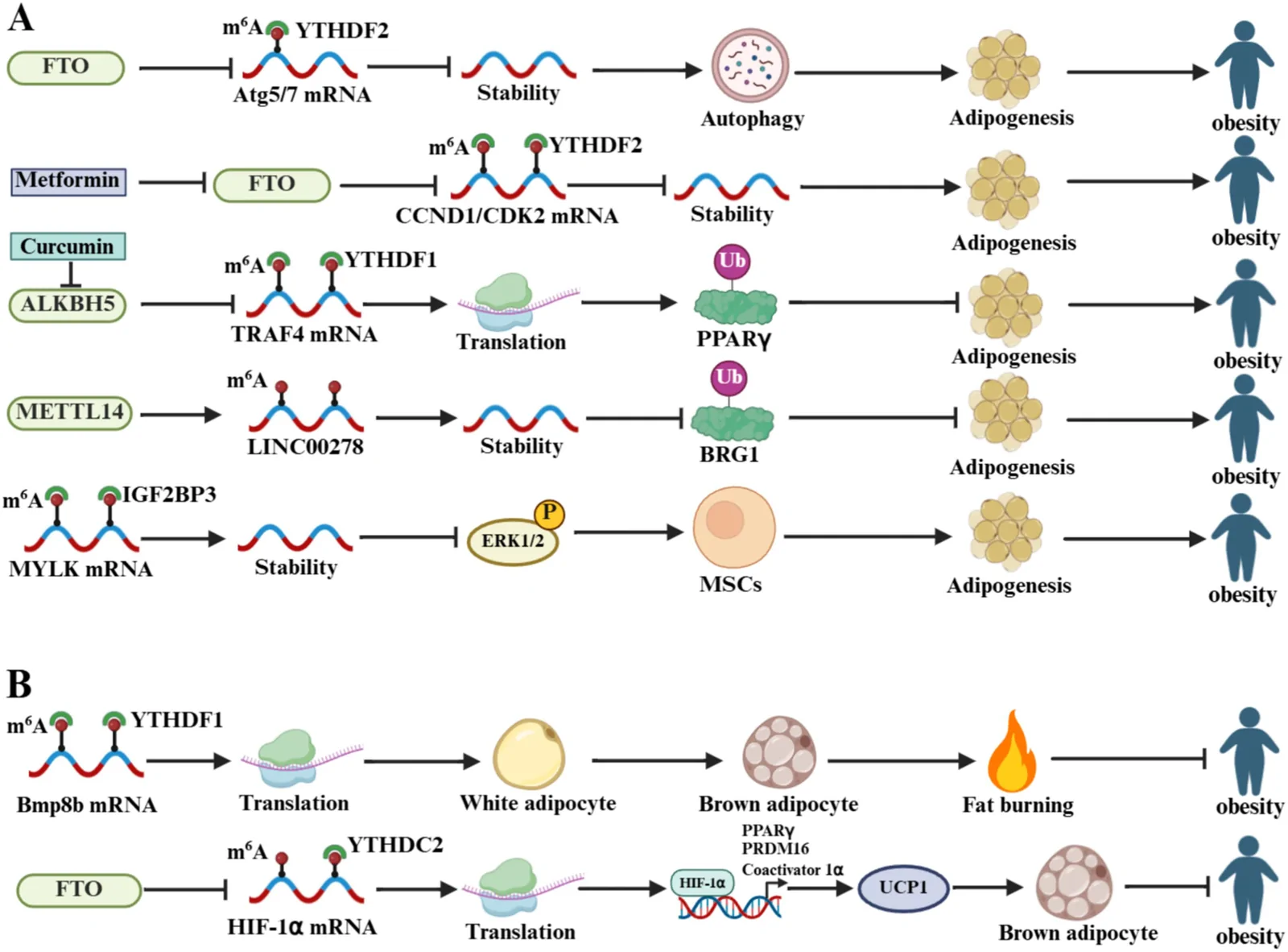
The role of m^6^A modification in obesity. **(A)** FTO works in concert with YTHDF2 to stabilize Atg5 and Atg7, promoting autophagy, adipogenesis and obesity. Metformin inhibits CCND1 and CDK2 expression and adipogenesis and combats obesity through the FTO/YTHDF2 pathway. Curcumin enhances TRAF4 translation by inhibiting ALKHB5/YTHDF1-mediated m^6^A demethylation, which promotes PPARγ protein degradation as well as alleviates adipogenesis and obesity. METTL14 promotes adipogenesis and obesity through the LINC00278/BRG1 pathway. IGF2BP3 suppresses the transformation of MSCs into adipocytes and obesity through the MYLK/ERK1/2 pathway. **(B)** YTHDF1 promotes Bmp8b translation and brown adipose tissue formation, leading to alleviation of obesity. Additionally, FTO/YTHDC2-mediated m^6^A demethylation decreases HIF-1α mRNA translation, which inhibits the transcription of coactivator 1α, PRDM16 and PPARγ, down-regulates UCP1 expression, inhibits white-to-brown fat transition and aggravates obesity.

**FIGURE 6:**
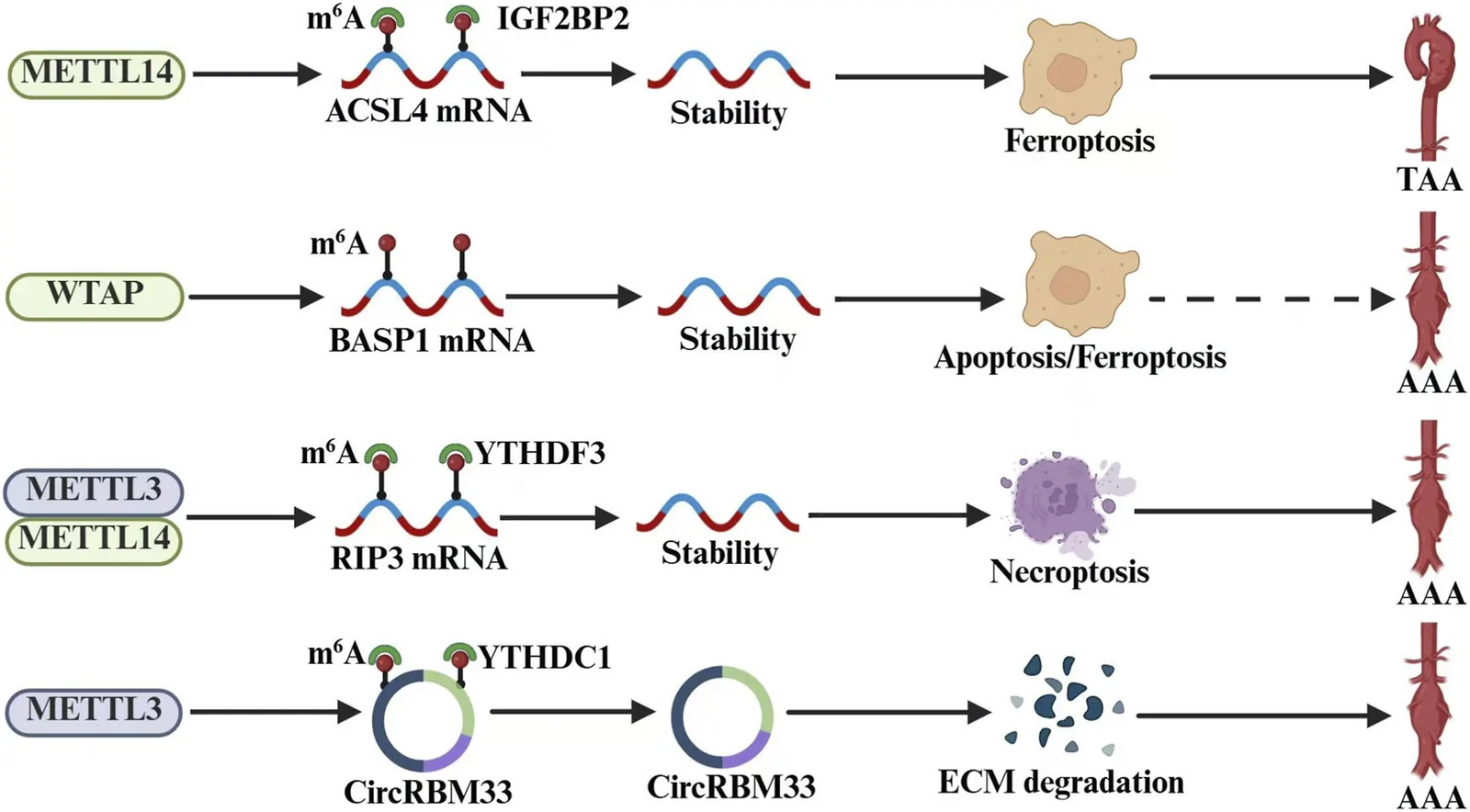
The m^6^A modification affects aortic aneurysm progression through multiple mechanisms. The m^6^A modification mediated by METTL14/IGF2BP2 enhances ACSL4 mRNA stabilization, promotes VSMC ferroptosis and TAA development. WTAP-induced m^6^A methylation stabilizes BASP1 to facilitate VSMC apoptosis and ferroptosis. The METTL3/METTL14 complex works in concert with YTHDF3 to increase RIP3 mRNA stability and promote VSMC necroptosis and AAA progression. METTL3 catalyzes m^6^A modification of circRBM33 and promotes its biogenesis by subsequent YTHDC1 recognition, accelerating ECM degradation and AAA.

### 5.1 Hypertension

Hypertension is a common cardiovascular disease resulting from the complex interaction between genetic and environmental factors and causes the damage of multiple target organs, such as heart, brain and kidney. Hypertension not only places increased pressure on the aortic wall contributing to the development of an intimal tear, but also stimulates pro-inflammatory cytokine release to trigger endothelial dysfunction (Konukoglu and Uzun, 2017). In addition, hypertension promotes the generation of matrix metalloproteinases, leading to excessive ECM degradation that compromises the elastic properties of the wall (Yin and Pickering, 2016). Approximately 80% of patients who develop an AD are estimated to have hypertension (Landenhed et al., 2015). Therefore, the control of hypertension is essential for decreasing the risk of AD.

Dysregulation of m^6^A modification is closely relevant to the pathophysiology of hypertension. It was found that METTL3 expression and global m^6^A levels are significantly increased in the arteries of hypertensive patients and Ang II-treated mice (Fei et al., 2025). Moreover, METTL3-mediated m^6^A methylation of a disintegrin and metalloproteases 17 (ADAM17) mRNA promotes its nuclear export, which in turn cleaves angiotensin converting enzyme 2 (ACE2), reduces Ang-(1–7) production, promotes vasoconstriction and increases blood pressure in Ang II-induced hypertensive mice (Fei et al., 2025). This reveals METTL3 as a contributor to hypertension. Another study showed that deletion of maternal METTL3 improves vascular dysfunction, alleviates vascular inflammation, and reduces susceptibility to hypertension in the adult mouse offspring (Zheng et al., 2025). This indicates that METTL3-mediated m^6^A modification plays an important role in the transgenerational transmission of maternal hypertension. The rostral ventrolateral medulla is located in the brainstem and functions a critical regulator of blood pressure. The expression levels of YTHDF3 are higher in the rostral ventrolateral medulla of spontaneously hypertensive rats than in Wistar Kyoto rats (Zhang et al., 2025b). YTHDF3 knockdown in the rostral ventrolateral medulla of spontaneously hypertensive rats suppresses neuronal apoptosis and reduces sympathetic tone and blood pressure, which is attributed to increased mRNA stability of X-ray cross complementing protein 1 (XRCC1) in an m^6^A-dependent manner (Zhang et al., 2025b). These findings derived from human observational data, *in vitro* experiments and animal models shed light on the underlying mechanism of hypertension from the perspective of m^6^A modification, providing potential targets for therapeutic interventions.

### 5.2 Atherosclerosis

Atherosclerosis, a chronic vascular pathology occurring large- and middle-size arteries, constitutes the major pathological basis of cardiovascular diseases such as myocardial infarction and stroke. Notably, atherosclerosis also serves as a significant risk factor for AD. Epidemiological and clinical studies have demonstrated that approximately 31% of AD patients have atherosclerosis (Pape et al., 2015).

Inflammatory response and lipid metabolism disorder are two critical events in the development of atherosclerosis. The relationship of m^6^A with these two pathological processes has attracted a lot of attention. For instance, the expression of METTL3 in macrophages increases with the progression of atherosclerosis (Li et al., 2023b). Macrophage-specific METTL3 deletion in mice reduces the binding of YTHDF1 to m^6^A-modified sites on B-Raf proto-oncogene (BRAF) mRNA and subsequent translation efficiency, leading to inhibition of extracellular signal-regulated kinase 1/2 (ERK1/2) phosphorylation and inflammatory response and subsequent alleviation of atherosclerosis (Li et al., 2023b). Similarly, METTL3-mediated m^6^A modification significantly increases lncRNA H19 stability through subsequent IGF2BP2 recognition, which stimulates EC pyroptosis and promotes atherosclerotic plaque formation (Tang et al., 2024a). METTL14 catalyzes the m^6^A modification of forkhead box O1 (FOXO1) mRNA, which is recognized by YTHDF1 to enhance its translation efficiency, thereby stimulating the transcription of vascular cell adhesion molecule-1 (VCAM-1) and intercellular adhesion molecule-1 (ICAM-1) and aggravating endothelial inflammation and atherosclerosis (Jian et al., 2020). ATP binding cassette transporter A1 (ABCA1) is an integral membrane protein and plays a central role in mediating intracellular cholesterol efflux. Studies from our group and others have demonstrated that promoting ABCA1 expression can suppress the formation of foam cells, the hallmark of early fatty streak lesions during atherogenesis (Zhao et al., 2021; Xue et al., 2022). A recent study showed that METTL3 cooperates with YTHDF2 to down-regulates zinc finger protein 771 (ZNF771) expression in a m^6^A-dependent, which inhibit the liver X receptor α (LXRα)/ABCA1 signaling pathway and thus promotes macrophage-derived foam cell formation and atherosclerosis development in mice (Tu et al., 2026). In addition, Yang et al. reported that METTL14 cooperates with YTHDF1 to enhance m^6^A methylation and stabilization of ubiquitin carboxyl-terminal hydrolase L5 (UCHL5) mRNA, which inhibits the ubiquitination of NLRP3, activates NLRP3 inflammasome and exacerbates atherosclerosis (Yang et al., 2023c). Nuclear paraspeckle assembly transcript 1 (NEAT1) is a recently discovered lncRNA that is widely expressed in a variety of mammalian cell types. Exercise can down-regulate METTL14 expression, inhibit endothelial pyroptosis and delay atherosclerosis (Yang et al., 2023b). Mechanistic studies showed that the m^6^A modification of NEAT1 mediated by METTL14/YTHDC1 facilitates its export from the nucleus to the cytoplasm, and then NEAT1 binds to Kruppel-like factor 4 (KLF4) to promote the transcriptional activation of NLRP3 (Yang et al., 2023b). These findings obtained from *in vitro* experiments and animal models offer a basis for targeting METTL3 and METTL14 molecules as well as their downstream pathways to slow down atherosclerosis progression.

Dysregulation of gut microbiota and their metabolites is involved in the onset and development of atherosclerosis (Fusco et al., 2026). As a recently identified gut microbiota-derived metabolite, 3-hydroxystearic acid (C18-3OH) has a significant effect on glucose, lipid and energy metabolism (Moens de Hase et al., 2023). Administration of C18-3OH represses lipid accumulation in macrophages and attenuates atherosclerosis in mcie (Zhou et al., 2026). Mechanistically, C18-3OH enhances paired box protein 8 (PAX-8) expression by inhibiting ALKBH5-mediated m^6^A demethylation, thereby facilitating ABCA1 transcription and subsequent cholesterol efflux from macrophages (Zhou et al., 2026). Cellular senescence is a state of permanent cell cycle arrest which is accompanied by halted proliferation triggered by endogenous or exogenous stimuli and plays an important role in atherogenesis (Wang and Bennett, 2012). Of note, ALKBH5 knockout disrupts the stability of CC chemokine ligand 5 (CCL5) mRNA stability by increasing m^6^A methylation, which down-regulates CCL5 expression, promotes autophagy, inhibits senescent macrophage formation, and diminishes atherosclerotic plaque area with greater stability (Gao et al., 2025). Collectively, these *in vitro and in vivo* data suggest that ALKBH5 plays a causative role in atherosclerosis and represents a novel therapeutic target.

### 5.3 Obesity

Obesity is a complex metabolic disorder that is defined as a body mass index of 30 kg/m^2^ or above for adults. At present, about 650 million adults as well as 340 million children and adolescence suffer from obesity (Sorensen et al., 2022). With the rapid changes in dietary structure and lifestyle, the incidence of obesity displays an increased trend, posing a big threat for human health. Extensive research has established a strong association between obesity and multiple metabolic disorders, including cardiovascular diseases, type 2 diabetes mellitus, and metabolic dysfunction-associated fatty liver disease (Koenen et al., 2021; Huttasch et al., 2024). It is worth noting that like hypertension and atherosclerosis, obesity is also a well-recognized risk factor for AD (Yu et al., 2024; Zhang et al., 2025a). Thus, maintaining a normal body weight is important to inhibit the formation and rupture of AD.

Adipogenesis plays a central role in the onset and development of obesity. It has been demonstrated that silencing of FTO enhances the m^6^A methylation of autophagy related 5 (Atg5) and Atg7 transcripts and promotes their degradation by recruiting YTHDF2, thereby alleviating autophagy, adipogenesis, and obesity (Wang et al., 2020). Metformin is the most commonly prescribed drug for type 2 diabetes mellitus. Administration of metformin reduces mitotic clonal expansion, inhibits the differentiation of preadipocytes into adipocytes and protects against high-fat diet-induced obesity in mice (Liao et al., 2022). Further study revealed that metformin down-regulates FTO expression and increases m^6^A methylation levels of cyclin D1 (CCND1) and cyclin dependent kinase 2 (CDK2), with YTHDF2 recognizing the m^6^A-modified sites and promoting the decay of CCND1 and CDK2 (Liao et al., 2022). Curcumin, a natural polyphenolic compound extracted from turmeric, possesses multiple pharmacological activities and has a protective effect on metabolic diseases (Jabczyk et al., 2021). Treatment with curcumin inhibits ALKHB5 expression, leading to increased m^6^A methylation of TNF receptor-associated factor 4 (TRAF4) mRNA. Subsequently, YTHDF1 combines with m^6^A-modified sites and enhances TRAF4 translation, which promotes ubiquitin proteasome system-mediated degradation of peroxisome proliferator-activated receptor γ (PPARγ) and alleviates adipogenesis and obesity (Chen et al., 2021). In addition, METTL14 expression is significantly increased in visceral adipose tissues from obese individuals compared with lean controls, and exhibits a positive correlation with body mass index (Zhang et al., 2025c). METTL14-mediated m^6^A methylation of LINC00278 enhances its stability of LINC00278, which inhibits the ubiquitination degradation of brahma-related gene-1 (BRG1), facilitates preadipocyte differentiation and increases body weight in C57BL/6 J mice (Zhang et al., 2025c). Adipocytes are known to originate from mesenchymal stem cells (MSCs). Overexpression of IGF2BP3 was found to repress the differentiation of MSCs into adipocytes and reduce body weight in high-fat diet mice (Huang et al., 2024). Mechanistically, IGF2BP3 interacts with the m^6^A site of myosin light chain kinase (MYLK) mRNA to extend its half-life and inhibit ERK1/2 phosphorylation (Huang et al., 2024). Taken together, the m^6^A-related agents finely modulate adipogenesis through various approaches and mechanisms although they play different roles in this process, thereby providing essential insights into the molecular regulatory mechanisms of obesity.

In general, adipose tissue is divided into three types: white adipose tissue, brown adipose tissue and beige adipose tissue. White adipose tissue can be converted to brown adipose tissue for generating heat through uncoupled oxidative phosphorylation, representing a promising strategy to combat obesity (Cheng et al., 2021). It has been demonstrated that acute exercise can promote the browning of white adipose tissue in an m^6^A-dependent manner (Chen et al., 2025). The expression of YTHDF1 is significantly decreased in adipose tissue of male mice fed a high-fat diet (Yan et al., 2023). Moreover, white adipose tissue-specific overexpression of YTHDF1 in mice increases the translation efficiency of bone morphogenetic protein 8b (Bmp8b) in an m^6^A-dependent manner, leading to induction of the browning process and alleviation of obesity (Yan et al., 2023). In contrast, FTO-mediated m^6^A demethylation inhibits white-to-brown fat transition, reduces energy expenditure and predisposes mice to obesity (Wu et al., 2021). Mechanistically, FTO knockout elevates the m^6^A levels of hypoxia-inducible factor-1α (HIF-1α) mRNA, which is recognized by YTHDC2, enhancing HIF-1α mRNA translation and expression. Subsequently, HIF-1α promotes the transcription of coactivator 1α, PR domain containing 16 (PRDM16) and PPARγ, which cooperatively upregulate uncoupling protein 1 (UCP1) expression and stimulate the browning of white adipocytes (Wu et al., 2021). In summary, these functional studies performed in cellular experiments and animal models highlight m^6^A as an important regulator for the conversion of white adipose tissue to brown adipose tissue. Nevertheless, whether the promotive effect of ALKHB5 on obesity is also associated with this process remains unclear and needs to be investigated.

### 5.4 Aortic aneurysm

As a chronic disease, aortic aneurysm refers to a permanent, irreversible and localized enlargement of the aorta. According to the anatomical location, it is classified as thoracic aortic aneurysm (TAA), abdominal aortic aneurysm (AAA), and thoracic-abdominal aortic aneurysm. The majority of patients are usually asymptomatic except when aortic aneurysm rupture. At present, there are no effective drugs to suppress the growth or rupture of aortic aneurysm. Surgical intervention or endovascular repair is the only option for the treatment of this condition. Unless treated immediately, aortic aneurysm is reported to be fatal in 50%–80% of cases, posing a significant threat to human life (Kuivaniemi et al., 2015). The presence of an aortic aneurysm has been shown to be associated with incremental risk of AD at the level of the diseased segment (Kuang et al., 2016). Patients with aortic aneurysm of >60 mm in diameter have a yearly rate of rupture or dissection of ≥6.9% and a death rate of 11.8% (Nienaber et al., 2016).

VSMCs play a critical role in regulating vessel tone and blood pressure as well as in maintaining vascular homeostasis. The loss of VSMCs caused by apoptosis and ferroptosis can weaken aortic wall and enlarge blood vessel, ultimately leading to the development of aortic aneurysm (Chen et al., 2022a). In the aortic tissue samples of human and mouse aortic aneurysm, global m^6^A methylation levels are significantly increased and show a close association with abdominal aortic aneurysm progression (He et al., 2019; Chen et al., 2024). Consistently, YTHDF3 is highly expressed in aortic tissue of AAA patients and positively correlates with aneurysm diameter (Chen et al., 2026). Furthermore, YTHDF3 restrains the proliferation but facilitates apoptosis in Ang II-treated VSMCs (Chen et al., 2026). Acyl-CoA synthase long-chain family member 4 (ACSL4) is a key isoenzyme involving ferroptosis. METTL14-mediated m^6^A modification of ACSL4 mRNA can increase its stability and subsequent expression by recruiting IGF2BP2, thereby promoting VSMC ferroptosis and aggravating TAA (Wang et al., 2025c). WTAP was shown to facilitate Ang-II-induced VSMC apoptosis and ferroptosis by enhancing the stability of brain abundant membrane attached signal protein 1 (BASP1) mRNA in an m^6^A-dependent manner (Tian et al., 2025). In addition, Li et al. reported that METTL3 forms a complex with METTL14 to stimulate VSMC necroptosis and inflammatory response as well as exacerbate AAA (Li et al., 2023a). Mechanistic studies further revealed that the METTL3/METTL14 complex catalyzes the m^6^A methylation of receptor-interacting protein 3 (RIP3) transcript, which is recognized by YTHDF3 to inhibit its degradation, leading to upregulation of RIP3 expression (Li et al., 2023a). These *in vitro* and *in vivo* findings indicate that m^6^A-associated factors play an important regulatory role in VSMC death during aortic aneurysm, offering potential targets for disease diagnosis and treatment.

ECM degradation can increase aortic wall fragility and have been recognized as a contributor to aortic aneurysm (Rabkin, 2017). CircRNAs are a class of single-stranded RNA molecules with covalently linked head-to-tail topology. Dysregulation of circRNAs has been proven to participate in the onset and development of aortic aneurysm (Liu et al., 2021; He et al., 2022). As a recently discovered circRNA, circRBM33 is generated from the exons of pre-mRNA of RNA binding motif protein 33 (RBM33) by backsplicing. It has been reported that circRBM33 expression is significantly increased in aortic tissue of AAA patients, and its overexpression sponges miR-4268 to enhance of EPH receptor B2 expression and degrade ECM in VSMCs challenged by Ang-II (Wang et al., 2021b). Notably, METTL3 cooperates with YTHDC1 to promote circRBM33 biogenesis in an m^6^A-dependent manner, resulting in ECM degradation and AAA development (Xu Y. et al., 2024). Thus, METTL3-mediated m^6^A methylation could be a potential candidate for the treatment of aortic aneurysm.

## 6 The diagnostic and therapeutic potential of m^6^A modification in AD

Because AD can lead to a poor outcome, rapid diagnosis and decision making are great importance to improve the prognosis of AD patients. The diagnosis of AD primarily depends on imaging techniques such as CT and MRI. Currently, some biomarkers, such as D-dimer, soluble ST2 and aggrecan, are being evaluated as novel methods for detecting AD (Wang et al., 2018; Konig et al., 2021; Yao et al., 2021). Recently, Guo et al. analyzed the expression pattern of m^6^A regulators in healthy and AD samples by using the GEO database and found that WTAP, HNRNPC and FTO are significantly upregulated while IGF2BP1 is down-regulated in AD samples (Guo et al., 2022). The AD diagnostic model further revealed that both FTO and IGF2BP1 exhibit a relatively excellent accuracy in predicting AD (Guo et al., 2022). Thus, FTO and IGF2BP1 could be developed as the potential biomarkers for the diagnosis of AD. Of note, current evidence supporting diagnostic applications of FTO, IGF2BP1 or other m^6^A regulators remains preliminary.

As mentioned above, m^6^A plays a key role in the pathogenesis of AD and has a significant effect on its major risk factors. Thus, regulating m^6^A-related molecules and downstream pathways represents a promising approach for developing new therapeutic interventions for AD. Currently, a variety of synthetic small-molecule inhibitors of m^6^A writers and erasers have been identified. STM2457 is a highly potent and selective first-in-class catalytic inhibitor of METTL3, with a direct interaction with the METTL3/METTL14 heterodimer for competition on m^6^A-containing substrate binding (Yankova et al., 2021). Prevention of METTL3 by STM2457 has been reported to suppress macrophage activation, pro-inflammatory cytokine release and atherosclerotic plaque formation by decreasing m^6^A methylation of lysine demethylase 6B (Huangfu et al., 2025). Similarly, administration of STM2457 significantly reverses m^6^A dysregulation and alleviates pulmonary arterial hypertension (He et al., 2025). Additionally, RSM3 and KH12 are able to promote METTL3 degradation to attenuate its activity and global RNA methylation levels, thereby providing a mechanism that distinct from other small-molecule competitive inhibitors of METTL3 (Hwang et al., 2024; Li et al., 2024b). 5-Carboxy-8-hydroxyquinoline (IOX1) is identified as an inhibitor of ALKBH5. Treatment with IOX1 attenuates macrophage senescence and protects against atherosclerosis by inhibiting ALKBH5-mediated m^6^A demethylation of CCL5 mRNA (Gao et al., 2025). Given that both METTL3 and ALKBH5 function as critical contributors to AD (Wang et al., 2021a; Zhu et al., 2021), application of these small-molecule inhibitors against m^6^A may offer an opportunity for the management of AD. Further studies are required to verify their efficacy and safety in animal models and early-phase clinical trials.

Natural compounds constitute another major class of m^6^A regulators. Coptisine chloride is a natural small-molecule and serves as a novel inhibitor of METTL3. A recent experimental study has shown that administration of coptisine chloride up-regulates TNF-α-induced protein 3 (TNFAIP3) expression, suppresses NLRP3 inflammasome activation and alleviates inflammatory response (Zhou et al., 2024). Mechanistically, coptisine chloride inhibits METTL3-mediated TNFAIP3 mRNA m^6^A modification to enhance its stability, which promotes the ubiquitination and degradation of NIMA-related kinase 7 and ultimately impairs NLRP3 inflammasome assembly (Zhou et al., 2024). Curcumin, a natural compound extracted from the root of the turmeric plant, has been proven to slow down the progression of AD (Liu S. et al., 2025). Interestingly, curcumin was found to down-regulate METTL3 expression and diminish the m^6^A abundance of circ0030568, leading to its destabilization (Sun et al., 2024). Hence, it is likely that the protective effect of curcumin on AD is associated with its ability to hinder METTL3 expression. This possibility needs to be confirmed in future research.

MiRNAs are a class of endogenous non-coding RNA molecules with a length of about 20–25 nucleotides. Mature miRNAs can inhibit gene expression by promoting target mRNA degradation or suppressing the translation process (Vishnoi and Rani, 2023). Overexpression of miR-145 restrains the transformation of VSMCs from contractile phenotype to synthetic phenotype and consequently protects against AD (Qiu et al., 2021). Also, dysregulation of miR-186-5p is involved in AD progression (Hu et al., 2025). Of note, YTHDF2 and METTL3 have been identified as the direct target genes of miR-145 and miR-186-5p, respectively (Li et al., 2020; Ma et al., 2024). Thus, stimulation of endogenous expression or exogenous delivery of these miRNA mimics could be valuable for altering m^6^A methylation and treating AD.

Despite METTL3 inhibitors, ALKBH5 inhibitors, natural compounds and miRNA-based approaches could hold significant promise for developing new therapies in AD, none of these approaches has been evaluated specifically in clinically relevant AD models. Thus, these strategies remain largely preclinical, and several critical challenges should be addressed before clinical translation. First, considering that a variety of RNA molecules can be modified by m^6^A, the effectiveness, specificity and safety of m^6^A-targeted epigenetic interventions for AD should be carefully evaluated. Second, limitations in delivery technologies constitute a major barrier. Systemic administration may cause off-target effects, which can disrupt physiological m^6^A methylation status and induce transcriptomic instability in non-target cells, tissues or organs. This may lead to tissue damage and interfere with energy supply and metabolic balance in normal cells. Thus, there is an urgent need to develop precise targeted delivery strategies. A potential strategy is to utilize engineering nanocarriers, such as liposomes and exosomes, for precise delivery to lesioned aorta. Third, since there is a significant cross-talk among various RNA methylation modifications (Tu et al., 2025), regulation of m^6^A alone may affect other modification types and consequently cause unexpected results. This cannot be ignored. Finally, the feasibility, efficacy and safety of combination therapies with m^6^A-targeting strategies and conventional therapeutic methods need to be assessed in animal models and clinical experiments. For instance, whether integrating METTL3 inhibitors with blood pressure lowering drugs, such as esmolol, can improve therapeutic outcomes through synergistic multi-pathway modulation is required to be addressed in the future.

## 7 Conclusion and future directions

The m^6^A methylation is regarded as the most prevalent and abundant mRNA modification in eukaryotic cells, exists in almost all RNA molecules, and plays a fundamental role in modulating RNA metabolism. The m^6^A-associated factors including writers, erasers and readers are commonly expressed abnormally in AD. Importantly, m^6^A plays a promotive or protective role in AD formation and progression depending on the context. This not only provides a novel perspective to deeply explore the pathogenesis of AD in the future, but also offers promising epigenetic targets for therapeutic interventions.

The dysregulated m^6^A modification has expanded our understanding about epigenetic regulation in the pathogenesis of AD. Nevertheless, the investigation of m^6^A in AD remains in its infancy, and there are still a lot of issues that need to be addressed. Current studies predominantly focus on the role of METTL3, KIAA1429, RBM15 and ALKBH5 in AD. Future research should explore the influence of other m^6^A-cytalyzed enzymes, such as METTL5, METTL14, METTL17, PCIF1 and FTO, on the influence of AD progression. Angiogenesis refers to the growth of new capillaries from the existing blood vessels. Aortic angiogenesis is believed to a characteristic pathological change of AD, because it can promote ECM remodeling, inflammatory cell infiltration and AD development (Jia et al., 2023; Wu et al., 2025). There is increasing evidence that m^6^A plays an important role in regulating neovascularization (Sun et al., 2026; Wei et al., 2026). However, it remains unknown whether m^6^A methylation modification exerts an effect on neovessel formation in the context of AD, which deserves further investigation. AD can be managed by open surgical repair, minimally invasive endovascular intervention or drugs. Future work should explore the feasibility of the strategy aiming to combine m^6^A with these therapeutic methods. Finally, it is of great importance to develop predictive biomarkers based on m^6^A regulator expression or global m^6^A levels in blood samples of AD patients. This may contribute to early disease diagnosis and risk stratification as well as help monitor therapeutic efficacy.

In summary, the investigation of m^6^A methylation in AD is currently undergoing rapid development. Although many issues remain to be resolved in future research, continued efforts in this field offer hope for translational medicine. The progress in this area will not only broaden our understanding of RNA epigenetic modulation but also offer new insights into the potential biomarkers and therapeutic approaches for AD.
